# An *in vitro* model of *Mycobacterium leprae* induced granuloma formation

**DOI:** 10.1186/1471-2334-13-279

**Published:** 2013-06-20

**Authors:** Hongsheng Wang, Yumi Maeda, Yasuo Fukutomi, Masahiko Makino

**Affiliations:** 1 Institute of Dermatology, Chinese Academy of Medical Sciences and Peking Union Medical College, 12 Jiangwangmiao Road, Nanjing 210042, China; 2 Department of Mycobacteriology, Leprosy Research Center, National Institute of Infectious Diseases, 4-2-1 Aobacho, Higashimurayama, Tokyo 189-0002, Japan

**Keywords:** Mycobacteria, Leprosy, Granuloma

## Abstract

**Background:**

Leprosy is a contagious and chronic systemic granulomatous disease caused by *Mycobacterium leprae*. In the pathogenesis of leprosy, granulomas play a key role, however, the mechanisms of the formation and maintenance of *M. leprae* granulomas are still not clearly understood.

**Methods:**

To better understand the molecular physiology of *M. leprae* granulomas and the interaction between the bacilli and human host cells, we developed an *in vitro* model of human granulomas, which mimicked the in vivo granulomas of leprosy. Macrophages were differentiated from human monocytes, and infected with *M. leprae*, and then cultured with autologous human peripheral blood mononuclear cells (PBMCs).

**Results:**

Robust granuloma-like aggregates were obtained only when the *M. leprae* infected macrophages were co-cultured with PBMCs. Histological examination showed *M. leprae* within the cytoplasmic center of the multinucleated giant cells, and these bacilli were metabolically active. Macrophages of both M1 and M2 types co-existed in the granuloma like aggregates. There was a strong relationship between the formation of granulomas and changes in the expression levels of cell surface antigens on macrophages, cytokine production and the macrophage polarization. The viability of *M. leprae* isolated from granulomas indicated that the formation of host cell aggregates benefited the host, but the bacilli also remained metabolically active.

**Conclusions:**

A simple *in vitro* model of human *M. leprae* granulomas was established using human monocyte-derived macrophages and PBMCs. This system may be useful to unravel the mechanisms of disease progression, and subsequently develop methods to control leprosy.

## Background

Leprosy is a chronic mycobacterial infection that presents an extraordinary range of cellular immune responses in humans. Regulation of cell-mediated immunity against *Mycobacterium leprae* through the fine-tuning between cells, cytokines and chemokines continues to be unraveled. Similar to other mycobacterial infections, granulomatous inflammation in the skin lesion defines certain forms of leprosy [1,2]. The bacilli enter and replicate within macrophages, resulting in the production of cytokines and chemokines, which in turn triggers an inflammatory response leading to the recruitment of macrophages and lymphocytes at the infectious site. Granulomas mainly contain macrophages, epithelioid cells (ECs), multinucleated giant cells (MGCs), surrounded by a rim of T lymphocytes [3]. The organization and the cellular constituents of the developing *M. leprae* granulomas vary with the status of the host immune response. Presumptively, granulomatous lesions can be categorized within two polar forms [4]. At one extreme, tuberculoid granulomas are organized as nodular lesions with ECs and MGCs in the lesion center surrounded by a rim of fibrous connective tissue, lymphocytes along the periphery of the granuloma, and acid-fast bacilli are rarely demonstrable in the lesions. At the other extreme, the pathological feature of lepromatous leprosy skin lesions are characterized by a lack of organization of cells, with very high numbers of foamy macrophages containing very large numbers of bacilli, and disorganized lymphocyte infiltration.

Granulomas have long been believed to benefit the host by containing and restricting the growth of mycobacteria in a localized area, to prevent the spread of the disease to other parts of the tissue or organs [5]. However, some studies in zebra fish infected with *M. marinum and M. tuberculosis* suggested that the granulomas contribute to early bacterial growth and expanding infection [6-10].

The structure, function, and evolution of granulomas have been studied using various animal models [11,12], high-resolution chest computed tomography scans of pulmonary tuberculosis patients [13], and explanted tissues [5,14]. Interestingly, the *in vitro* models of human mycobacterial granulomas have been studied by infection with Bacillus Calmette-Guérin (BCG) or stimulation with antigens such as purified protein derivatives or artificial beads coated with mycobacterial components [15,16]. These studies have identified infected macrophages, ECs, and several types of MGCs, which are thought to play important roles in the formation and maintenance of granulomas. In addition, macrophages demonstrate considerable plasticity that allows them to efficiently respond to environmental signals. These cells are generally classified as M1 (classic) macrophages, which produce proinflammatory cytokines and mediate resistance to pathogens and contribute to tissue destruction, or M2 (alternative) macrophages, that produce anti-inflammatory cytokines and promote tissue repair [17-19]. However, so far, we know little about the relationship between the polarization of macrophages within mycobacterial granulomas.

In this study, we developed an *in vitro* model of *M. leprae* granulomas, which mimicked the human granulomatous skin lesion with progressive recruitment of monocytes around macrophages infected by *M. leprae*, and their differentiation into ECs and MGCs as well as recruitment of activated lymphocytes. This model may be useful for unravelling the mechanisms of disease progression, and find effective strategies to control the spread of bacilli.

## Methods

### Ethics statement, cell culture and preparation of the bacteria

Peripheral blood was obtained from healthy Japanese individuals with informed consent. The study was approved by the ethics committee of the National Institute of Infectious Diseases (NIID). In Japan, BCG vaccination is compulsory for children aged 0–4 years old. Macrophages were differentiated from monocytes using granulocyte-macrophage colony-stimulating factor (GM-CSF) as described previously [20,21]. Animal experiments were carried out in strict accordance with the recommendations of Japan’s Animal Protection Law. The protocol was approved by the Experimental Animal Committee of NIID Tokyo (Permit Number: 211002). *M. leprae* (Thai-53 strain) was propagated in athymic BALB/c-*nu*/*nu* mice (Clea Co, Tokyo) [22]. At 8–9 months post-infection, mouse footpads were processed to recover *M. leprae*[23]. For all experiments, *M. leprae* was freshly prepared. Human cells without the bacilli were cultured at 37°C but when infected with the bacilli, the cells were cultured at 35°C to maintain the viability of *M. leprae* in host cells.

### Culture of macrophages and peripheral blood mononuclear cells for the formation of cellular aggregates

Macrophages, differentiated from monocytes using GM-CSF after 4 days culture in RPMI containing 20% fetal calf serum (FCS) were transferred into 24-well tissue culture plates (Falcon) (1 ~ 2 × 10^5^ cells/well). Freshly prepared *M. leprae* were then added to each well. The multiplicity of infection (MOI: 50) was determined based on the assumption that macrophage were equally susceptible to infection with *M. leprae*[24]. After 24 hr, autologous peripheral blood mononuclear cells (PBMCs) were cultured with *M. leprae* infected macrophages at a ratio of 5:1 (PBMCs:macrophages). In some cases, macrophages were infected with *M. leprae* without PBMCs and in others, macrophages and PBMCs were co-cultured and macrophages alone were used as negative controls. The cells were cultured at 35°C for periods from 24 h to 10 days with medium changes every other day. To detach the cells from plates TrypLE Express (Gibco) was used, and then the cells were maintained in medium containing 10%FCS for 30 min, before processing for flow cytometric analyses. In other experiments we have also isolated T lymphocytes and monocytes were isolated using Dynabeads Untouched Human T cells and Dynabeads MyPure Monocyte kit 2 (Invitrogen), and used instead of PBMCs.

### Phase-contrast microscopy and fluorescence microscopy

Macrophages grown on a 13-mm coverslip in a 24-well plate, were infected with *M. leprae* for 24 h. Autologous PBMCs were then co-cultured with macrophages for additional 9 days. Macrophages were fixed in 2% paraformaldehyde, or methanol pre-chilled to −20°C, and then observed under a phase-contrast microscope (Olympus CKX41 with × 10 and × 20 objective lenses). Photographs were taken with an Olympus DP50 system. Image acquisition and data processing were performed using DP controller software. In other experiments, cells were stained with May-Grünwald-Giemsa stain (MGG) (Sigma-Aldrich) or by TB Carbolfuchsin ZN stain according to the manufacturer’s instructions (BD Biosciences).

Cell imaging was performed using LSM5-Exciter laser scanning microscope equipped with a 568 nm laser (Carl Zeiss). Fixed cells were stained with anti-human CD163 monoclonal antibody (mAb: BioLegend) and the secondary antibody used was an Alexa Fluor 568-conjugated goat anti-mouse IgG (Invitrogen/Molecular Probes). Nuclei were counterstained with Hoechst 33342 dye (Sigma-Aldrich). *M. leprae* was stained by auramine O (BD Biosciences). Images were obtained under a fluorescence confocal microscope. Data were processed using LSM software ZEN 2007.

### Analysis of cell surface antigens on macrophages by flow cytometry and microscopy

Macrophages were collected after time points of 1 and 9 days of co-culture with the PBMCs or *M. leprae* stimulation. The expression of cell surface antigens on macrophages, was analyzed using a FACSCalibur flow cytometer (BD Biosciences). Dead cells were eliminated from the analysis by staining with 7-amino actinomycin D. For the analysis of cell surface antigens, the following mAb were used: FITC-conjugated mAb against CD68 (KP) was purchased from Dako, FITC conjugated TLR4 (HTA125) and CD206 (19.2), and PE conjugated mAb against CD86 (FUN-1) was all purchased from BD Biosciences and PE conjugated mAb to CD14 (HCD14) and CD163 (RM3/1) were from BioLegend. The numbers in the insets indicate the mean fluorescent values of the cells stained with the respective mAbs.

### Determination of cytokine levels

The levels of the cytokines: Interferon (IFN)-γ, interleukin (IL)-2, tumor necrosis factor (TNF)-α, IL-12p40, IL-1β and IL-10 in the culture supernatants were quantified using enzyme assay kits, OptEIA Human ELISA Set (BD Biosciences) and processed according to the manufacturer’s instructions. IL-4 and IL-13 was purchased from MABTECH AB. Cytokine levels were expressed as pg of protein/ml of protein. Real-time PCR analysis of mRNA extracted using an RNeasy Mini kit (Qiagen), was performed using SYBR Green PCR Master Mix (Applied Biosystems) with specific primers according to the manufacturer’s instructions. The instrument used for the detection of the expression of mRNA was StepOnePlus with StepOne software.

### Determination of *M. leprae* viability

The viability of *M. leprae* recovered from the macrophages of different groups was detected by radiorespirometry, that measures the oxidation of ^14^C palmitic acid to ^14^CO_2_, as described previously [25]. Briefly, the adherent macrophages and granulomas with bacilli were lysed in 300 μl of a 0.1 N NaOH solution to release intracellular *M. leprae*. After neutralization with 0.1 N HCl solution, an equal volume of 2 times concentrated Middlebrook 7H9 broth was added. ^14^C labeled palmitic acid was added to the lysates of macrophages or granulomas, followed by incubation at 33°C. After 7 days, cumulative amounts of oxidized palmitic acid released as ^14^CO_2_ by metabolically active *M. leprae* were measured using a Packard 1500 TRI-CARB liquid scintillation analyzer. The unpaired Student’s t-test was used to determine the statistical significance of the two data sets.

## Results

### Granuloma-like aggregates formed by co-culture of *M. leprae* infected macrophages and autologous PBMCs

**Figure 1:**
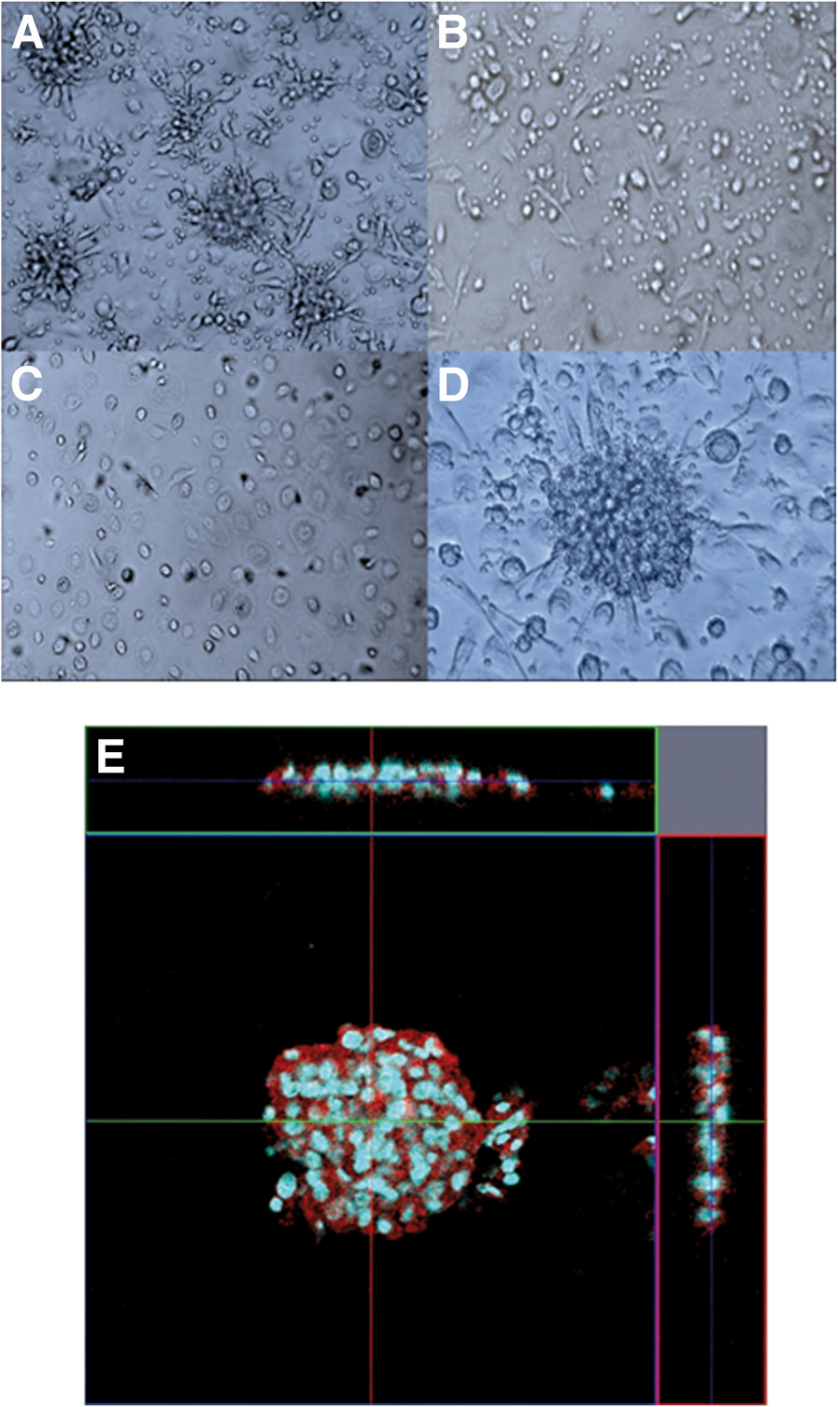
**Formation of granuloma-like cellular aggregates by co-culture of PBMCs and macrophages infected with *M. leprae*.** (**A**) Co-culture of macrophages (1 × 10^5^), infected with *M. leprae* (MOI:50) and autologous PBMCs (5 × 10^5^) in a 24 well-plate resulted in the formation of granuloma-like aggregates by day 9. (**B**) Culture of macrophages (1 × 10^5^) and autologous PBMCs (5 × 10^5^) for 9 days, without the bacilli. No formation of granuloma-like aggregates was observed. (**C**) Macrophages (1 × 10^5^) infected with *M. leprae* (MOI:50) after 9 days co-culture. (**D**) Higher magnification (2×) of the cell-aggregates in (A). (**E**) Confocal microscopic (LSM5 Exciter) analysis of *M. leprae*-induced granuloma revealed a multilayered structure (about 3–4 cell layers cells in transverse and straight sections). The cells in aggregates were positive for CD163 (red), a marker of macrophages. Nuclei were stained with Hoechst 33343 (blue). Representative data from a single donor are shown.

When PBMCs were incubated with *M. leprae* infected macrophages in a 24-well tissue culture plate, the cells aggregated to form a multilayered granuloma-like aggregates by day 9 as shown in Figure 1A, whereas control groups did not recruit any cells at this stage (Figure 1B, C). We observed formation of a granular ball-like structure caused by some synapses around aggregates. These *in vitro* granulomas exhibited a cellular structure similar to that in histopathological specimens of tuberculoid leprosy lesions showing T lymphocytes surrounding the differentiated, ECs and MGCs that may be involved in cytokine production for intercellular communication. (Figure 1D). Confocal microscopic analysis of *M. leprae*-induced granuloma showed a multilayered structure (about 3–4 cell layers in transverse and straight sections), and some cells were positive for CD163 (red), a macrophage marker (Figure 1E).

### Characterization of the cell populations recruited within *in vitro* granuloma-like aggregates

**Figure 2:**
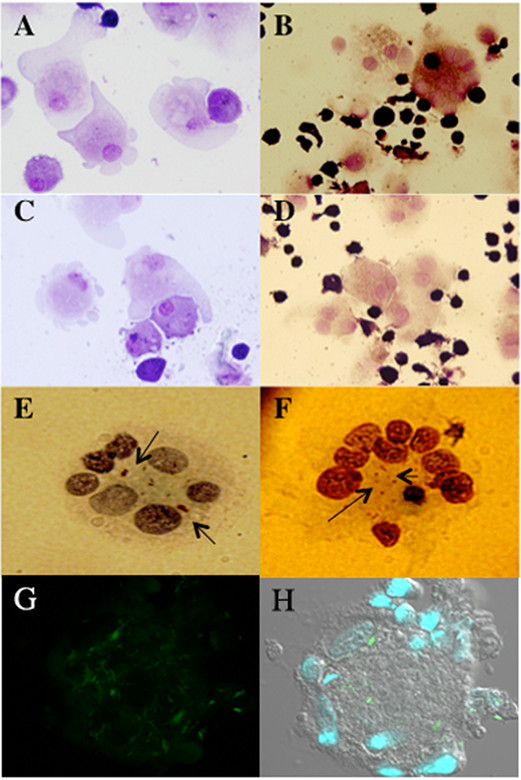
**Cell populations in granuloma-like aggregates.** May–Grünwald–Giemsa (MGG) staining showed that there are mainly macrophages, ECs (**A**, **D**) and MGCs in the aggregates (**B**, **D**). MGCs were formed by the intercellular fusion and phagocytosis of cells (**C**). *M. leprae* were stained with Ziehl-Neelsen (shown with arrows) and the bacilli were found to be restricted to the central cytoplasmic region of the MGCs (**E**, **F**). Confocal microscopy of MGCs showed *M. leprae* stained with auramine O (green) and the nuclei stained with Hoescht (**G**, **H**).

To identify and characterize the different cell types in granuloma-like aggregates, the cells were plated on glass slides and stained on day 9 of co-culture. MGG staining showed that activated macrophages with larger cytoplasm, and MGCs were observed, which resembled those in the granulomas of leprosy (Figure 2B, D). MGCs are thought to be formed as a result of fusion of macrophages, monocytes and ECs (Figures 2A, C). The presence of *M. leprae* in MGCs was confirmed by staining with TB Carbolfuchsin ZN (arrows in Figure 2E, F). In addition, confocal microscopy revealed the presence of MGCs with auramine O stained *M. leprae*, in the cytoplasmic region (Figure 2G, H). To characterize macrophages, ECs and MGCs in the granuloma-like aggregates, we performed immunofluorescence staining for macrophage markers CD68, CD1a and CD163 (data not shown). Both the macrophages and the MGCs could express the CD68 and CD1a marker, but the expression level of CD68 on the macrophages was higher than that on the MGCs. With the increasing number of nuclei in MGCs, lower levels of CD68 was observed (not shown), although there was no significant difference in the expression levels of CD1a between macrophages and MGCs. These data indicate that MGCs belong to the monocyte/macrophage lineage.

### Expression levels of cell surface antigens on macrophages at different time points

**Figure 3:**
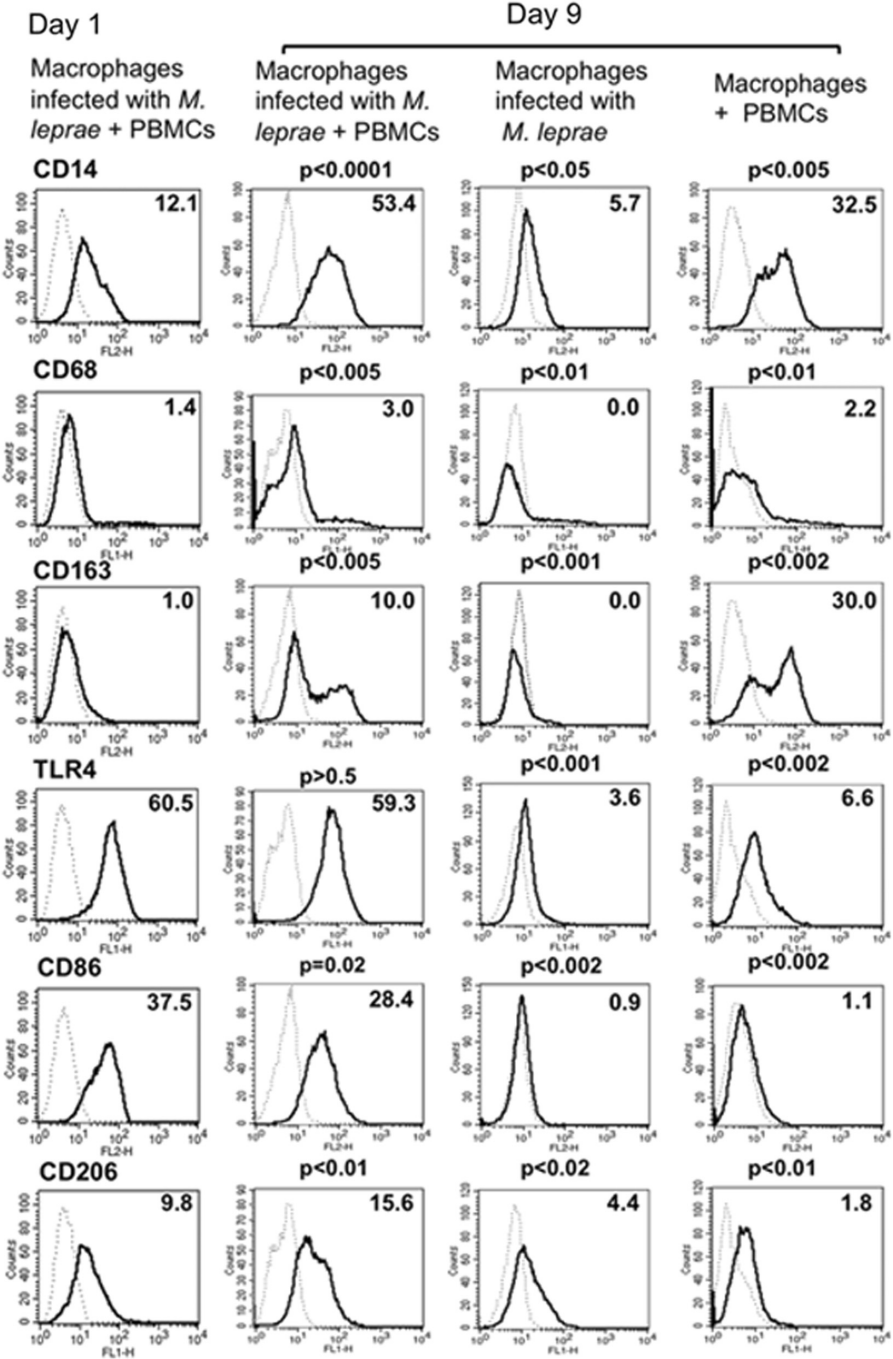
**Expression of cell surface antigens on macrophages at two different time points.** Compared with the control group on day 1, day 9 macrophages infected with *M. leprae* and co-cultured with T lymphocytes showed relatively higher expression of CD14, CD163 and CD206. While in macrophages infected with *M. leprae*, the expression level of CD14, CD68, CD163, TLR4, CD86 and CD206, were downregulated as compared to those infected macrophages co-cultured with PBMCs. Representative data of one donor, from three independent experiments are shown. P-values were calculated using the Welch unpaired t-test in comparison with day 1 macrophages.

We investigated the expression levels of cell surface antigens on macrophages from different groups at two different time points, day 1 and day 9. On day 1, there was no significant difference in the expression of cell surface antigens on macrophages between groups. Compared with day 1 macrophages, day 9 macrophages, which were infected with *M. leprae* and co-cultured with PBMCs to form granuloma-like aggregates, showed higher expression of CD14 (pattern recognition receptor), CD68 (macrophage marker related to phagocytic activities), CD163 (scavenger receptor) and CD206 (mannose receptor), although the expression of major histocompatibility complex (MHC) class-II, CD86, and toll-like receptor (TLR)-4 did not change (Figure 3). Interestingly, in our long-term culture (9 days) of macrophages infected with *M. leprae*, the expression of CD14, CD68, CD163, TLR4, CD86 and CD206 was significantly lower than that in macrophages infected with *M. leprae* and co-cultured with PBMCs. CD206 expression was the lowest in macrophages co-cultured with PBMCs, although CD163 expression was significantly high (Figure 3). CD163 and CD206 are markers of M2 macrophages, whereas CD86 expression is associated with M1 macrophages. Therefore, the M1 and M2 macrophages appeared to coexist in granulomas.

### Cytokines in culture supernatants

**Figure 4:**
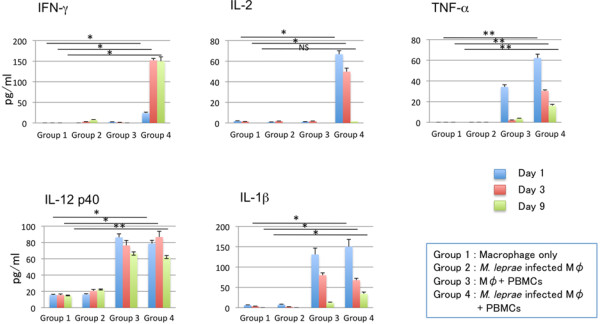
**Measurement of cytokines secreted into the culture medium by ELISA.** Measurement of IFN-γ, IL-2, TNF-α, IL-12, and IL-1β secreted in the culture medium from different groups of cells at day 1, 3 and 9. Representative data from three individual experiments of a single donor are shown. Unpaired Student’s t-test was performed. NS: not significant, *p < 0.001, **p < 0.01.

**Figure 5:**
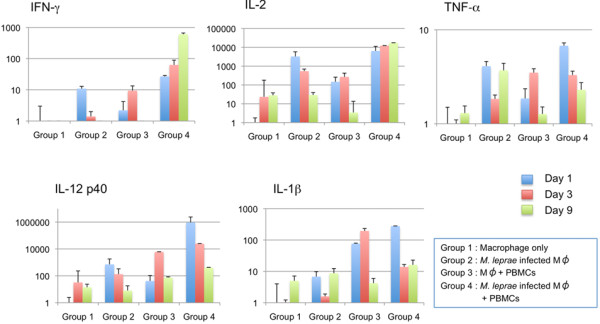
**Determination of mRNA level of cytokines by real-time PCR.** mRNA expression of IFN-γ, IL-2, TNF-α, IL-12, and IL-1β in the cells was evaluated by using specific primers, SYBR Green PCR Master Mix, and a StepOnePlus (Applied Biosystems). Cells were collected on days 1, 3 and 9 after co-culture with or without PBMCs. The y-axis shows the relative quantitative values calculated from comparative Ct values normalized to the control (actin used as calibrator). The standard deviation of the ΔCt value was determined (Applied Biosystems). Representative data from a single donor of three individual experiments are shown.

The culture supernatants from different groups were collected on days 1, 3 and 9 after the start of macrophage culture. The release of IFN-γ, IL-2, TNF-α, IL-12p40, IL-1β, IL-4 and IL-13, was evaluated by ELISA (Figure 4). Interestingly, the expression levels of the various cytokines in supernatants, from different groups showed significant differences that were associated with the formation of granuloma-like aggregates and changes of cell surface antigen expression on macrophages. In the group with *M. leprae* infected macrophages co-cultured with PBMCs, the concentrations of IL-2, IL-1β and TNF-α peaked on day 1 after infection and then declined gradually. The level of IL-12 p40 also declined slowly by day 9. IFN-γ levels were low on day 1, but increased 7 fold by day 4, and then remained unchanged till day 9. A high level of IL-10 expression in macrophages and macrophages cultured with PBMCs was observed, but the expression was significantly decreased when macrophages were infected with *M. leprae* as observed in the day 9 cytokine expression levels. However, when macrophages were differentiated with M-CSF, the expression of IL-10 was significantly high when macrophages were infected with *M. leprae* (Additional file 1: Figure S1). IL-4 and IL-13 were not detected in any groups on days 1 and 9 from the start of macrophage culture (data not shown). Real time PCR results further confirmed the cytokine expression and showed similar results except for the IL-2 and TNF-α, whose expression was observed in control groups of macrophages infected with *M. leprae* in addition to those co-cultured with PBMCs (Figure 5).

### The viability of *M. leprae* in granuloma-like aggregates

**Figure 6:**
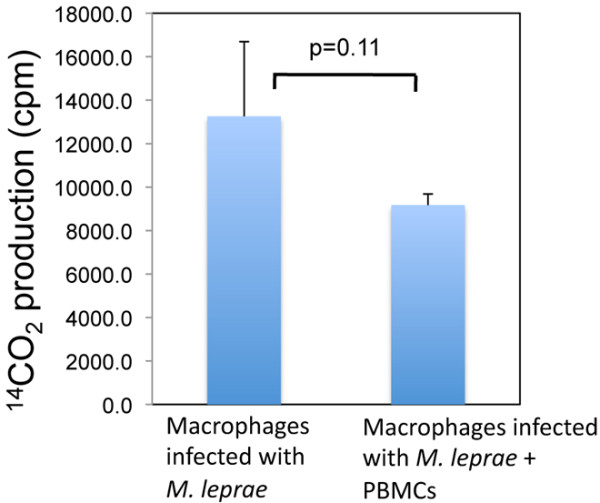
**Measurement of the viability of *M. leprae *in macrophages after co-culture with PBMCs.** At day 9 after culture of macrophages infected *M. leprae* with or without PBMCs, macrophages were lysed, and the viability of *M. leprae* was determined by a radiorespirometric assay. Unpaired Student’s t test was used to determine the statistical significance of the two data sets. Representative data of three individual experiments are shown.

We determined the viability of *M. leprae* at days 1 and 9, when granuloma-like aggregates were observed in co-cultures of *M. leprae* infected macrophages with PBMCs, whereas in cultures of macrophages infected with *M. leprae*, there was no granuloma formation. The amount of radioactive CO_2_ evolved which reflects the rate of ^14^C-palmitic acid oxidized by *M. leprae*, which was measured by a scintillation counter. No significant difference in ^14^CO_2_ production was observed from macrophage in either groups on days 1, and 9. However, the amount of radioactive CO_2_ released from macrophages infected with *M. leprae* and co-cultured with PBMCs for 9 days was lower but not significantly lower than that released from macrophages infected with *M. leprae* alone (Figure 6).

## Discussion

In the 1960s, Ridley and Jopling proposed a histological classification scheme for leprosy [26]. At one extreme, called the polar tuberculoid, leprosy patients show a high degree of cell-mediated immunity, lesions revealing well-developed granulomatous inflammation and rarely acid-fast bacilli are detected. At the other extreme, termed polar lepromatous patients have no apparent resistance to *M. leprae*, and skin biopsies reveal sheets of foamy macrophages in the dermis containing very large numbers of bacilli and microcolonies called globi. Currently, the formation and maintenance of granulomas are considered to be critical components of the host response to *M. leprae* infection, which determine not only whether primary disease occurs, but also the clinical manifestation. Granuloma formation is studied in mouse models but little is known about the human granuloma due to the ethical problems of using human samples and the difficulties in establishing a good model using human cell lines.

The formation of small, rounded granuloma-like-structure, was previously described by co-culture of blood lymphocytes with autologous macrophages infected with *M. tuberculosis*, or BCG or stimulation with other mycobacterial antigen such as purified protein derivatives. These granuloma-like structures showed abundance of CD68 positive macrophages with small round lymphocytes scattered throughout the granuloma [15,16]. These models not only exhibit structural similarities to granulomas observed in human clinical specimens, but also show patterns of cell antigen expression and/or cytokine production that appear consistent with those observed in tuberculosis patients. However, the formation of granulomas in leprosy, involving *M. leprae* infection has not been previously studied in vitro. The only data available on granuloma formation of leprosy is from the immunological staining of biopsies of patients, and granulomas harvested from the footpads of athymic nude mice [27].

In our model, we first infected the immature human macrophages with *M. leprae*. To mimic the recruitment of additional PBMCs which would occur in vivo, autologous PBMCs were added after 24 h and cultured at 35°C, the optimal temperature for the growth of *M. leprae* and macrophages to be kept viable. Within 9 days of culture, macrophages and T lymphocytes gathered to form a granuloma-like aggregates with fused macrophages, appearing as multinucleated cells, and epitheloid macrophages tightly linked to surrounding macrophages and lymphocytes. However, in control groups, the formation of granuloma-like aggregates was not observed. When autologous T lymphocytes and monocytes were purified and used instead of PBMCs, a similar formation of granuloma like aggregates were observed, together with production of the same amounts of cytokines, indicating that T lymphocytes and monocytes are sufficient for the containment of *M. leprae* in granuloma like structures.

Electron microscopy studies indicated that the tuberculoid lesion had an appearance of a granulomatous response with a predominance of ECs and MGCs, and the mononuclear phagocytes which are surrounded by a mantle of lymphocytes [28]. In the present *in vitro* model of granulomas, MGCs were prominent, and resembled MGCs observed in a tuberculoid lesion. MGCs have been described by Langhans, but the function of these cells in the granuloma remains to be elucidated [29]. In this study, we observed not only Langhans giant cells (MGCs with a circular nuclear organization in contrast to the MGCs formed in response to a foreign body that lacks this kind of organization), but also the bacilli surrounded by nuclei and restricted to the central cytoplasmic region. Because this type of MGC is not observed in the normal mouse model, it is interesting to further focus on the formation, mechanism and function of such MGCs using human *in vitro* model or humanized mouse model as recently described by Heuts et al. [30]. The *in vitro* model of leprosy granulomas still needs to be investigated, and compared to that obtained using leprosy patients’ monocytes and T cells.

Macrophages function as control switches of the immune system, providing a balance between pro- and anti-inflammatory responses by developing into subsets of M1 or M2 activated macrophages. M1 macrophages are activated by type I cytokines such as IFN-γ and TNFα, Alternatively, activated M2 macrophages are subdivided further into M2a (activated by IL-4 or IL-13), M2b (activated by immune complexes in combination with IL-1β) and M2c (activated by IL-10 or glucocorticoids) [31]. M1 macrophages exhibit a potent microbicidal activity, and release IL-12, promoting strong Th1 immune responses. It is the M1 population that is thought to contribute to macrophage-mediated tissue injury [19,32]. In contrast, M2 macrophages support Th2-associated effector functions and exert a selective immunosuppressive activity. M2 macrophages also play a role in the resolution of inflammation through phagocytosis of apoptotic neutrophils, reduced production of pro-inflammatory cytokines, and increased synthesis of mediators that are important for tissue remodeling and wound repair. We investigated the contribution of the macrophage polarization, MGC formation and immune responses against *M. leprae* in granulomas, and found that there was a strong relationship between the formation of granuloma-like aggregates, the changes of cell surface antigen expression on macrophages, and the expression levels of various cytokines with the macrophage polarization. In *M. leprae* infected macrophages co-cultured with PBMCs, the concentrations of IL-2, IL-12 and TNF-α peaked at day 1, while, TLR4, CD86, and MHC molecules were highly expressed, indicating that most of the macrophages were of the M1 subset. At day 9, in the same group of infected macrophages co-cultured with PBMCs, the cells assembled and formed a multilayer, granuloma-like aggregates, and the macrophages not only highly expressed TLR4 and CD86, but also scavenger receptor (CD163) and mannose receptor (CD206) molecules. CD163 and CD206 are the markers of M2 macrophages. Therefore, the M1 and M2 macrophages coexisted in granuloma-like aggregates. Consistent with this observation, the levels of IL-1β, IL-2, IL-12 and IFN-γ were high in the culture medium, promoting the differentiation of macrophages into both M1 and M2 subsets. The protective cell mediated immune response is regulated by the cytokine equilibrium, while the tuberculoid pole is characterized by the presence of Th1 cytokines (IL-2, IFN-γ, TNF-α and IL-12), and lepromatous is characterized by type 2 cytokines (IL-4, IL-6 and IL-10) [33]. Because IL-10 is an immunosuppressive cytokine implicated in susceptibility to mycobacterial infection, we examined the expression of IL-10 in more detail. Indeed, the infection with *M. leprae* suppressed the production of IL-10. However, when macrophages were differentiated with M-CSF, rather than GM-CSF, *M. leprae* infection further enhanced IL-10 production. Our results indicate that the granuloma aggregates studied here, are similar to those observed in the tuberculoid form of leprosy. However, little is known about the type of cytokines that influence the formation of macrophages for containment of *M. leprae* in the granulomas during the pathogenesis of leprosy.

We also investigated the viability of *M. leprae* in macrophages at different time points. At day 9, a number of granuloma-like aggregates were observed in co-cultures of PBMCs with macrophages infected with *M. leprae*. However in macrophages infected with *M. leprae* without the PBMCs, granuloma-like aggregates were not observed. There were no significant differences in the viability of *M. leprae* in macrophage of different groups on day 1, but on day 9, the viability of *M. leprae* in the group that formed granuloma-like aggregates was slightly lower, although not significantly, than that of *M. leprae* in infected macrophages without PBMCs. Evidently, granuloma-like aggregates appear to benefit the host but the bacilli remained metabolically active. The mechanism of this phenomenon needs further in-depth analysis.

## Conclusions

In summary, we have developed for the first time a method to obtain *in vitro M. leprae* granulomas using human monocyte derived macrophages and PBMCs. Using this model, we obtained some basic information about the characteristics of *in vitro* granulomas. In addition, the viability of *M. leprae* in granuloma-like aggregates remained unaltered during the culture period. Effective strategies to allow the bacilli to succumb to the formation of granuloma may assist in the primary control of the infection.

## Abbreviations

DCs: Dendritic cells; PBMCs: Peripheral blood mononuclear cells; ECs: Epitheloid cells; MGCs: Multinucleated giant cells; BCG: Bacillus Calmette- Guérin.

## Competing interests

The authors declare that they have no competing interests.

## Authors’ contributions

HW, YM participated in the design of the study and carried out the cell culture experiments, YF carried out the confocal microscopic examination, and radio-respirometric assay. HW, YM, and MM were involved in the preparation of the manuscript. All authors have read and approved the final manuscript.

## Pre-publication history

The pre-publication history for this paper can be accessed here:

http://www.biomedcentral.com/1471-2334/13/279/prepub

## Supplementary Material

Additional file 1: Figure S1 Measurement of IL-10 secreted into the culture medium by ELISA. Measurement of IL-10 secreted in the culture medium from different groups of cells at days 2, 6 and 9 is shown. Two types of macrophages were used to analyze the data. (A) Macrophages differentiated using GM-CSF, and (B) macrophages differentiated from monocytes using M-CSF. Representative data from two individual experiments of a single donor are shown. Unpaired student’s t test was performed, *p < 0.0001, **p < 0.001, ***p < 0.05. Click here for file
