# Supplementary figures and images for: An in vitro model of Mycobacterium leprae induced granuloma formation
Source: BMC Infect Dis. 2013 Jun 20;13:279. doi: 10.1186/1471-2334-13-279 (PMC3693892; doi:10.1186/1471-2334-13-279)

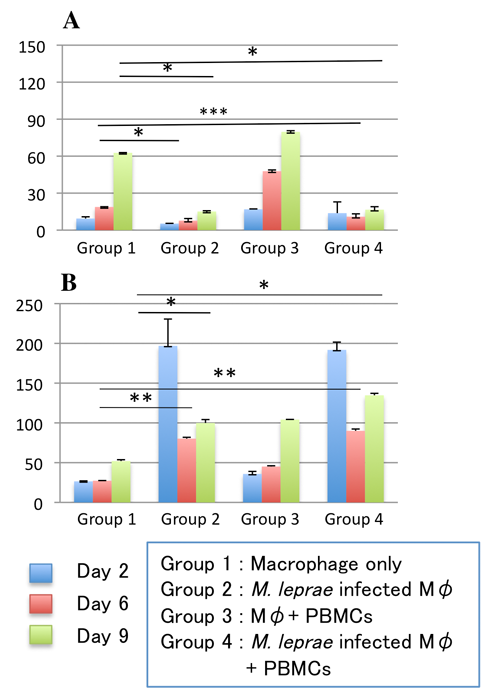

Supplement: Additional file 1: Figure S1 — Measurement of IL-10 secreted into the culture medium by ELISA. Measurement of IL-10 secreted in the culture medium from different groups of cells at days 2, 6 and 9 is shown. Two types of macrophages were used to analyze the data. (A) Macrophages differentiated using GM-CSF, and (B) macrophages differentiated from monocytes using M-CSF. Representative data from two individual experiments of a single donor are shown. Unpaired student’s t test was performed, *p < 0.0001, **p < 0.001, ***p < 0.05. [file 1471-2334-13-279-S1.tiff]
